# Language experience during the sensitive period narrows infants’ sensory encoding of lexical tones—Music intervention reverses it

**DOI:** 10.3389/fnhum.2022.941853

**Published:** 2022-07-09

**Authors:** Tian Christina Zhao, Fernando Llanos, Bharath Chandrasekaran, Patricia K. Kuhl

**Affiliations:** ^1^Institute for Learning & Brain Sciences, University of Washington, Seattle, WA, United States; ^2^Department of Speech and Hearing Sciences, University of Washington, Seattle, WA, United States; ^3^Department of Linguistics, University of Texas at Austin, Austin, TX, United States; ^4^Department of Communication Sciences and Disorders, University of Pittsburgh, Pittsburgh, PA, United States

**Keywords:** Infant speech learning, sensitive period, music intervention, speech encoding, frequency-following response (FFR), lexical tones

## Abstract

The *sensitive period* for phonetic learning (6∼12 months), evidenced by improved native speech processing and declined non-native speech processing, represents an early milestone in language acquisition. We examined the extent that sensory encoding of speech is altered by experience during this period by testing two hypotheses: (1) early sensory encoding of non-native speech declines as infants gain native-language experience, and (2) music intervention reverses this decline. We longitudinally measured the frequency-following response (FFR), a robust indicator of early sensory encoding along the auditory pathway, to a Mandarin lexical tone in 7- and 11-months-old monolingual English-learning infants. Infants received either no intervention (language-experience group) or music intervention (music-intervention group) randomly between FFR recordings. The language-experience group exhibited the expected decline in FFR pitch-tracking accuracy to the Mandarin tone, while the music-intervention group did not. Our results support both hypotheses and demonstrate that both language and music experiences alter infants’ speech encoding.

## Introduction

The *sensitive period* for phonetic learning occurs between 6 and 12 months of age and constitutes one of the earliest milestones in language acquisition, as the learning outcomes of this period have been shown to be reliable predictors of individual differences in later speech and language skills (Tsao et al., 2004; Kuhl et al., 2005, 2008; Zhao et al., 2021).

The main characteristic of the sensitive period is a “perceptual narrowing” phenomenon that occurs with increased native language experience. Specifically, infants’ ability to process non-native speech contrasts starts to decline, while infants simultaneously become significantly better at processing phonetic differences in their native language (Werker and Tees, 1984; Kuhl et al., 2006). Previously, Werker and Tees (1984) demonstrated a significantly reduced ability in English-learning infants to discriminate non-native Hindi and Salish consonant contrasts at 12 months, compared to infants at 6 months of age, while these contrasts remained highly discriminable for Hindi and Salish learning infants. These effects of linguistic experience on speech discrimination for non-native phonetic contrasts during this period have since been observed in both behavioral as well as neural responses to many phonetic contrasts (Kuhl et al., 1992, 2006).

Notably, this perceptual narrowing phenomenon has also been observed for lexical tones, which are linguistically relevant pitch patterns that contrast word meaning in more than half of the world’s languages (Hyman, 2001). For example, Mandarin Chinese has four distinctive lexical tones: /yi/ in Tone 1 (high-level pitch) means one, /yi/ in Tone 2 (low-rising pitch) means “aunt”, /yi/ in Tone 3 (low-dipping pitch) means “chair” and /yi/ in Tone 4 (high-falling tone) means “100 million.” Studies demonstrate that lexical tones are already more difficult to discriminate by 9 months of age for non-tonal language learning infants (Mattock and Burnham, 2006; Mattock et al., 2008).

A second characteristic of the sensitive period is the high malleability of its outcome, which has been demonstrated by interventions that provide infants with additional auditory experience (Kuhl et al., 2003; Zhao and Kuhl, 2017). In the seminal study, 9-month-old monolingual English-learning infants were randomly assigned to participate in 12 sessions of laboratory-controlled, live and highly interactive, exposure sessions with a foreign language speaker (Mandarin) vs. 12 sessions in English. After the intervention period, all infants were tested on their ability to discriminate a Mandarin contrast and only infants who completed the Mandarin sessions demonstrated ability to discriminate the speech contrast that was the same as Mandarin-learning infants (Kuhl et al., 2003). Critically, infants who completed Mandarin exposure sessions with only audio (i.e., listening to recordings) or audiovisual input (i.e., watching TV) did not demonstrate learning either, suggesting the crucial role of live interaction. This phenomenon has since been replicated with Spanish as well, with EEG measures characterizing neural sensitivity to speech contrasts (Conboy and Kuhl, 2011; Lytle et al., 2018). Taken together, intensive interactive foreign language interventions have been shown to reverse the decline in non-native speech discrimination during the sensitive period.

More recently, a study further demonstrated that a similar intervention with music (i.e., 12 interactive sessions starting at 9 months of age), instead of foreign language, was also able to enhance infants’ neural discrimination of a non-native speech contrast, in addition to enhancing neural processing of music rhythm (Zhao and Kuhl, 2016). This result was in line with a growing literature demonstrating a cross-domain generalization effect from early music training experience to speech processing, that is, an enhanced ability to process not only music, but also speech sounds, in adults and children who received significant music training early in life [e.g., (Pantev et al., 1998; Kraus et al., 2014)]. Critically, this study demonstrated such cross-domain generalization as early as in infancy. Together, these results support the hypotheses of shared neural mechanisms for speech and music that are underlying such generalization or transfer effects, such that strengthening the shared mechanisms through music is beneficial for speech processing as well (Patel, 2014).

In spite of these powerful demonstrations of changes in phonetic perception during the sensitive period, the neural mechanisms underpinning these experiential effects remain a topic of intense research interest (Kuhl, 2010). Previous EEG (Conboy and Kuhl, 2011; Lytle et al., 2018) and MEG (Kuhl et al., 2014; Zhao and Kuhl, 2016, 2022) studies have largely leveraged the mismatch negativity/response (MMN/MMR), generally considered to reflect higher-level neural processes that take place with much longer onset latency (e.g., onset after 150 ms) (Naatanen et al., 2007).

In contrast to previous studies, the current study focuses on the sensory encoding of speech that takes place with much shorter latencies, reflecting processing at much earlier stages along the auditory pathway, including the auditory brainstem. Particularly, these earlier auditory processing stages play a fundamental role in the sensory encoding or representation of acoustic attributes of speech, such as pitch. And the quality of such representation in the early stages of auditory processing are highly relevant for speech perception (Reetzke et al., 2018). Here, we examined the overarching question of whether early sensory encoding of speech is altered by experience during the sensitive period.

The measure used to assess early sensory encoding of speech is the frequency-following-response (FFR), a scalp recorded electrophysiological signal with short latency that robustly reflects phase-locking activity from multiple neural ensembles within the auditory system, especially at the earlier stages of processing (Coffey et al., 2019). The FFR provides an ideal means by which to evaluate the neural encoding of linguistically relevant, time-varying signals, such as pitch in lexical tones. Critically, the FFR has also been shown to be sensitive to an individual’s language and music experience in adults and older children (Skoe and Chandrasekaran, 2014). For example, tonal language speakers were observed to elicit FFRs that were better tracking the pitch of lexical tones than non-tonal language speakers (Krishnan et al., 2005; Bidelman et al., 2011). A recent study also showed that adults’ neural pitch tracking of lexical tones, as reflected in FFR, can be altered with even a short-term intensive laboratory-based training on lexical tones (Reetzke et al., 2018). Further, the generalization effect from music training to speech encoding was also observed in a study in which highly trained English-speaking adult musicians demonstrated better neural pitch tracking in their FFR to lexical tones than their non-musician counterparts (Wong et al., 2007).

The key question is thus whether auditory experience has an effect on sensory encoding of speech as reflected in the FFR, as early as in infancy during the sensitive period, or alternatively, whether changes only occur after a prolonged period of accumulation of auditory experience. There has been limited use of FFR to assess speech processing in infants (Lemos et al., 2021). In neonates, pitch tracking quality in FFR for lexical tones was observed to be distinct between infants born in China as compared to infants born the United States (Jeng et al., 2011, 2013). In older infants, enhancement have been documented for some aspects of FFR to native speech sounds, presumably reflecting developmental maturation of the auditory system (Anderson et al., 2015; Skoe et al., 2015; Musacchia et al., 2018; Novitskiy et al., 2021). No study that we are aware of has focused on the FFR during the sensitive period using longitudinal comparisons of infants with experimentally manipulated experiences to address this theoretical question.

**FIGURE 1:**
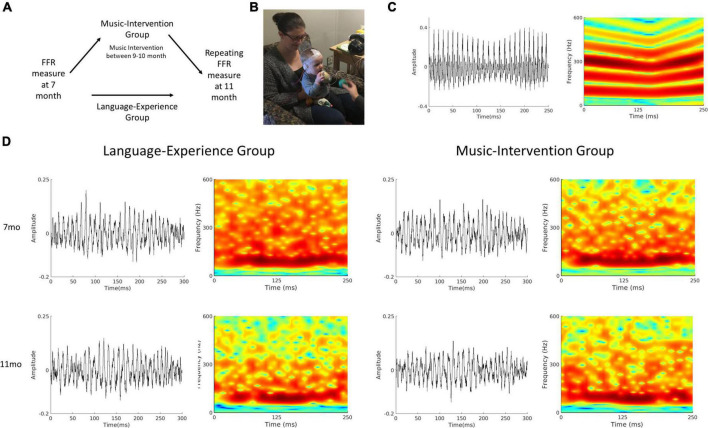
**(A)** Schematics of the experimental design. For the language-experience group, the same measurements were repeated at 11-month of age. The music-intervention group completed a 12-session music intervention starting at 9 months of age, in between the two FFR measurements. **(B)** A picture of an FFR recording session for a 7-month-old infant. **(C)** Waveform and spectrogram of the speech stimulus used for the FFR recording session. **(D)** Waveforms (left column) and spectrograms (right column) of the group-averaged FFR for the language-experience and the music intervention groups at 7- and 11-months of age.

In the current study we addressed this overarching key question by testing two specific hypotheses. First, we hypothesized that the perceptual narrowing phenomenon during the sensitive period can be detected in the early stages of sensory speech encoding. We tested this hypothesis by comparing sensory encoding, as reflected by FFR, of a non-native Mandarin Chinese lexical tone in English-learning infants measured longitudinally at 7 and 11 months of age. A second hypothesis was that the addition of music intervention during the sensitive period would reverse the perceptual narrowing effect for the non-native Mandarin tone. We tested this hypothesis by examining an additional group of infants who were randomly selected to receive a highly social music intervention in a controlled lab setting from 9 to 10 months of age, in between the two measurements of speech encoding (i.e., FFR) at 7- and 11- months of age (Figure 1A).

## Materials and methods

### Participants

Forty-three typically developing infants from monolingual English-speaking households were recruited at 24 weeks to participate in this longitudinal study. The inclusion criteria included the following: (1) full term and born within 14 days of the due date, (2) no known health problems and no more than 3 ear infections, (3) birth weight ranging from 6 to 10 lb., (4) no significant foreign language exposure (i.e., parents and regular caregivers speak only English to the infant) and (5) no previous or concurrent enrollment in infant music classes. All experimental procedures performed were in accordance with the Declaration of Helsinki and were approved by the University of Washington Institutional Review Board. All participating families gave informed consent and were compensated monetarily for their time and effort.

The semi-random assignment of group (music-intervention vs. language-experience) happened prior to recruitment by interspersing blocks of time that are dedicated to recruit for one group (e.g., one month recruiting for music-intervention group). Families were not aware of the other condition and were invited for either a 2-session or a 14-session study. For the language-experience group, of the 26 infants who were tested at 7 months of age (mean age = 27.27 weeks, SD = 0.94, N of male = 14), two became too fussy before data recording (i.e., refused placement of electrodes). Twenty infants returned for testing at 11 months of age (mean age = 48.56 weeks, SD = 1.22, N of male = 10). Of these, two become too fussy before data recording. For the music-intervention group, of the 17 infants that were tested at 7 months of age (mean age = 28.05 weeks, SD = 1.06, N of male = 9), 1 became too fussy before data recording (i.e., refused placement of electrodes), and one was not able to complete all 12 sessions of intervention. All other infants returned for testing at 11 months of age and were able to complete the session (mean age = 47.74 weeks, SD = 0.98, *N* of male = 8). Overall, the study was terminated early with a smaller N than the original target (*N* = 20 in each group) due to COVID-19 pandemic. The original target (*N* = 20) was determined based on an *a prior* power analysis with previous language and music intervention studies, using both behavioral and neural measures (Kuhl et al., 2003; Zhao and Kuhl, 2016), yielding power at 0.90 at a significant level of 0.05. This information was reported at clinicaltrials.gov under NCT04509739.

Infants’ backgrounds were surveyed through parental reports. For the language-experience group, at 7 months of age, the SES of the families averaged 53.64 (SD = 8.90) as measured by the Hollingshead scale. Ten of the 26 infants tested at 7 months of age were reported to have some level (Mean = 5.80 h/month, SD = 7.07) of foreign language exposure (i.e., music, library story time). Six were reported to live with an adult with significant music training (i.e., 8+ years of private lessons). On average, infants were estimated to have music exposure (i.e., parents singing, radio) for 20.57 h/week (SD = 13.44) at 7 months and an average of 26.35 h/per week (SD = 24.51) at 11 months. For the music-intervention group, at 7 months of age, the SES of the families averaged 51 (SD = 10.07) as measured by the Hollingshead scale. Ten of the 16 infants tested at 7 months of age were reported to have some level (Mean = 7.40 h/month, SD = 9.24) of foreign language exposure (i.e., music, library story time). Eight were reported to live with an adult with significant music training (i.e., 8 + years of private lessons). On average, infants were estimated to have music exposure (i.e., parents singing, radio) for 16.59 h/week (SD = 14.88) at 7 months and an average of 20.37 h/per week (SD = 15.05) at 11 months.

### Frequency-following response recording

#### Stimulus

**FIGURE 2:**
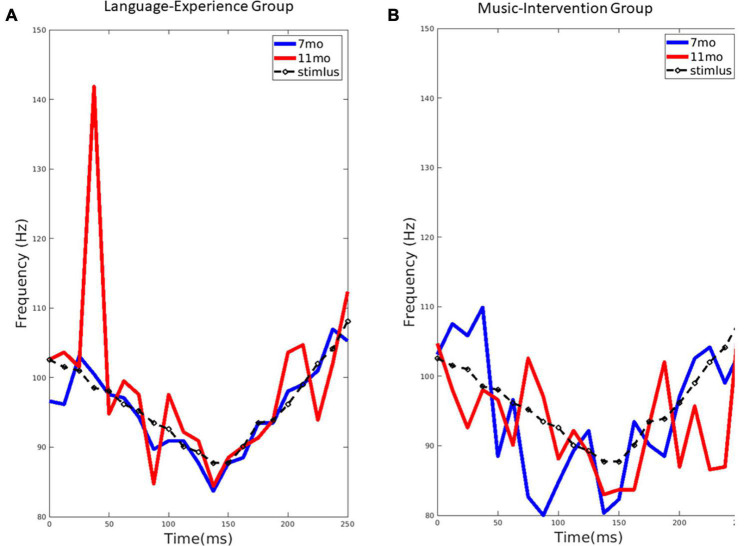
**(A)** The extracted pitch from the group-level FFR for the language-experience group at 7-month (blue line) and 11-month (red line), compared to the stimulus pitch contour (black dashed line). **(B)** The extracted pitch from the group-level FFR for the music-intervention group at 7-month (blue line) and 11-month (red line), compared to the stimulus pitch contour (black dashed line).

A synthesized high front vowel syllable /yi/ with Mandarin lexical tone 3 (dipping tone, meaning chair) was used as the non-native speech sound stimulus (Figure 1C). The stimulus was 250 ms in duration with a sampling frequency of 44,100 Hz. The fundamental frequency (*f0*) of the stimulus first fell from 103 to 89 Hz and then rose from 89 to 111 Hz (Figures 2A,B). This stimulus was selected because it has been previously demonstrated in adult studies to elicit the best frequency-following response out of the four Mandarin tones as well as the largest difference between native Mandarin speakers and English speakers (Llanos et al., 2017).

#### Materials and procedures

A NeuroScan system (Compumedics Inc., Melbourne, Australia) with a SymAmp^2TM^ Amplifier was used for recording the EEG signal. The stimulus was presented by the STIM2 software (Version 4.0, Audio CPT, Compumedics Inc., Melbourne, Australia), from a Dell Optiplex 755 computer to the Stim Audio System, and then monaurally through an insert earphone (NeuroScan) to the right ear at 75 dB with 3000 repetitions. The stimulus-onset-asynchrony (SOA) was jittered around 300 ms (±20 ms) and the polarity of the stimulus was alternated throughout the presentation. A conventional 3-electrode setup was adopted for the FFR recording (CZ, Ground at Forehead and Reference on the right ear lobe). Continuous EEG was amplified and recorded using the Scan software (Version 4.5, Compumedics Inc., Melbourne, Australia) at a sampling rate of 20 kHz.

The whole FFR recording session lasted approximately 45 min (∼15 min of recording). Upon the start of the session, the infant was seated comfortably in a caregiver’s lap. A research assistant used toys to maintain the infants’ calmness while an experimenter placed the electrodes on the infants’ scalp. Then the caregiver and infant moved into a sound-attenuated booth for the recording. The infant continued to be seated on the caregiver’s lap throughout the whole recording. The electrodes were attached to the EEG amplifier and were adjusted to achieve good impedance (<10 kΩ). The research assistant continued using quiet toys to maintain the infants’ calmness while the experimenter inserted and secured the earphone before starting the FFR recording (Figure 1B). Breaks were taken as needed when infants became fussy or when electrodes need to be adjusted. Recording stopped either when 3000 trials have been recorded or when infants became too fussy to continue.

For both groups of infants, the same FFR recording protocol was conducted twice, at 7-months and then repeated at 11 months of age.

### Music intervention

The music intervention methods repeated the protocols from the previous study (Zhao and Kuhl, 2016). Infants assigned to the music intervention group completed 12 sessions (15 min per session) of structured music intervention over a 4-week period starting at 9 months of age. The sessions took place in a sound-attenuating booth decorated to be infant-friendly. Recordings of children’s music in triple meter were selected from various commercially published music CDs for infants and toddlers. They were selected to vary in tempo (slow to fast; range, 115–180 beats per minute) and voices (for songs) to facilitate the musical activities and learning. All music was recorded on six CDs of about 15 min duration. In each session, one of six CDs was played through two speakers at a comfortable listening level of 65 decibels (A-weighted sound levels) (dBA), measured at the center of the room. Up to three infants and their primary caregivers were in the room, along with an experimenter who facilitated the session. The caregivers were instructed to interact with the infant throughout the sessions, with the aim of synchronizing the infants’ movements to the musical beats. A variety of infant-safe simple percussive musical toys were introduced to infants to facilitate infants’ movements, such as shaking maracas, foot tapping and bouncing.

### Data analyses

All data analyses were performed using EEGLAB (Delorme and Makeig, 2004) in combination with in-house scripts written with MATLAB R2020a (Mathworks, Natick, Massachusetts) software. The raw data was offline referenced to the Refence channel, band-pass filtered between 80 and 2000 Hz and then epoched between -50 and 300 ms in relation to stimulus onset. Trials with excessive noise (± 35mV) were rejected. Only recordings with a minimal of 1500 accepted trials were used in the subsequent analyses. In total, 16 infants in the language-experience group and 15 infants in the music-intervention group had accepted recordings at both 7 month and 11 month of age, and this dataset has been made publicly available on Open Science Framework (Zhao, 2022). The average number of accepted trials are 2380.90 (SD = 322.14) at 7 month and 2329.20 (SD = 382.21) at 11 month for the language-experience group, and 2188.20 (SD = 354.59) at 7 month and 2557.90 (SD = 340.72) at 11 month for the music-intervention group. Grand averaged FFR waveforms as well as spectrograms for both groups at both ages are visualized at Figure 1D.

Due to the low signal-to-noise ratio in individual infant FFR data for reliable *f0* extraction, the *f0* extraction was done on the group-averaged FFR and non-parametric permutation tests were used to statistically assess group-level differences for each comparison. To conduct the permutation tests, first, four group-level *observed* FFRs (i.e., language-experience group at 7 months, language-experience group at 11 months, music-intervention group at 7 months and music-intervention group at 11 months) were calculated by grand-averaging across all individual infant’s FFR within the corresponding group and age, with each individual FFR calculated by averaging across the first 1500 trials (750 trials from each polarity). For each *observed* group-level FFR, the fundamental frequency (*f0*) was then extracted using a sliding window (40 ms long with 30 ms overlaps) autocorrelation-based method to search for *f0* between 80 and 150 Hz, resulting in 21 pitch bins over the 250 ms segment (Xie et al., 2017).

We focused on FFR metrics that reflect the stimulus (i.e., pitch) tracking quality and calculated dependent measures from each group-level FFR. The main dependent measure was the *f0*-correlation, calculated as the Pearson correlation coefficient was between the *f0* extracted from the group-level FFR and the *f0* extract from the stimulus. Additionally, *f0-*error was calculated by summing the absolute difference between the stimulus *f0* from the FFR *f0* across all pitch bins. The *f0*-correlation and *f0*-error reflect the accuracy of pitch tracking, but for relative changes vs. absolute values of stimulus pitch. Third, *f0-*strength was indexed by the peak autocorrelation value for each pitch bin, indicating the robustness of neural phase-locking to the *f0* of the stimulus. In addition, we also calculated the signal-to-noise ratio (SNR) for each group-level FFR.

With the dependent measures, we conducted 3 statistical tests for our two main hypotheses. First, we tested that pitch tracking quality for the non-native Mandarin tone, as reflected by the FFR, would be lower at 11 months than at 7 months of age in the language-experience group, due to the expected perceptual narrowing effect. Then, we tested that the decline in pitch tracking quality, as measured with the FFR, would be reversed in the music-intervention group. Lastly, we tested that the changes between ages in pitch tracking quality are different between the language-experience group and the music-intervention group.

**FIGURE 3:**
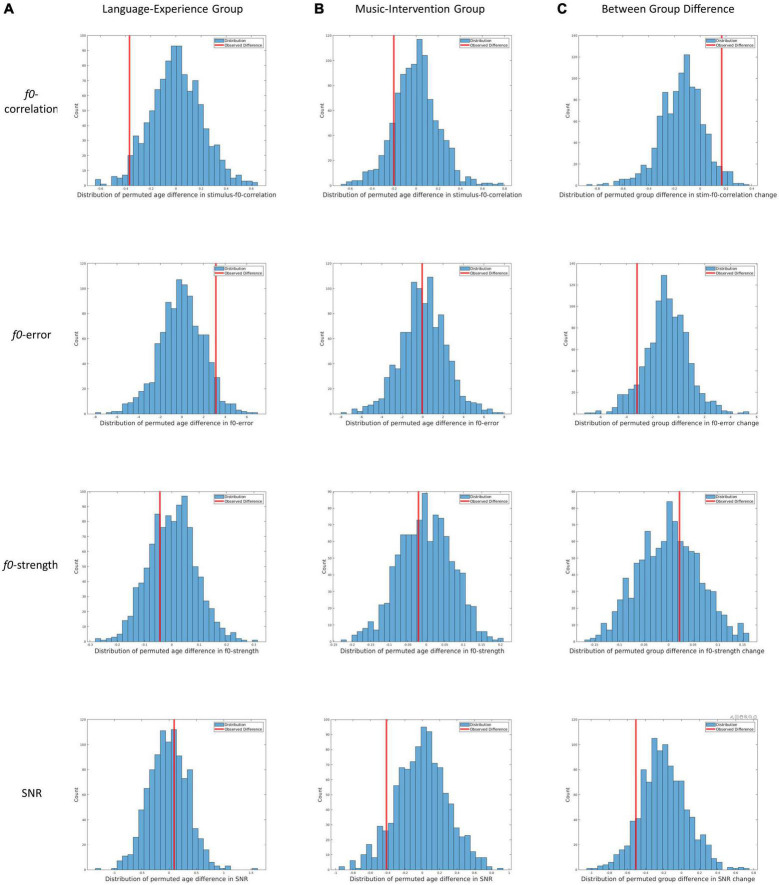
Results from the three permutation tests for all four dependent measures. **(A)** Distribution of permutated between-age differences in the language-experience group for *f0*-correlation, *f0*-error, *f0*-strength and SNR. Redlines indicate the observed between-age differences. **(B)** Distribution of permutated between-age differences in the music-intervention group for *f0*-correlation, *f0*-error, *f0*-strength and SNR. Redlines indicate the observed between-age differences. **(C)** Distribution of permutated between-group differences for change in *f0*-correlation, *f0*-error, *f0*-strength and SNR. Redlines indicate the observed between-group difference.

To test our first statistical hypothesis of whether pitch tracking quality declined from 7 to 11 months in the language-experience group, individuals’ FFRs from the language-experience group at both ages were first shuffled and randomly assigned to a “permuted 7-month group” and a “permuted 11-month group.” Then, for the two permuted age groups, group-level FFRs were calculated, and the same measures were extracted to quantify pitch tracking quality (i.e., *f0*-correlation etc.). A between-age difference was then calculated for each measure (i.e., *f0*-correlation for the permuted 11-month group – *f0*-correlation for the permuted 7-month group). This process was repeated 1000 times and distributions were built for each measure demonstrating the permuted differences between 7 and 11 months of age for the language-experience group (Figure 3A). Critically, the observed difference (difference between observed 7 months and 11 months measured in the language-experience group) was compared against this permuted distribution and an Achieved Significance Level (ASL) can be calculated as the proportion in the permuted distribution where the permutated difference is more extreme than the observed difference.

To test our second statistical hypothesis of whether decline in pitch tracking quality from 7 to 11 months is reserved in the music-intervention group, the same permutation test was conducted to evaluate whether there is a difference between 7-month and 11-month group-level FFR in the music-intervention group.

Lastly, to test our third statistical hypothesis of whether there is a between-group difference in the change of pitch tracking quality from 7 to 11 months, a slightly different permutation procedure was adopted. Particularly, all individuals from both groups were randomly assigned into a “permuted language-experience group” or a “permuted music-intervention group.” Then, for each permuted group, the two group-level FFRs (i.e., one for 7 months and one for 11 months) were first calculated by using the corresponding data. The same measures were extracted, and between-age differences were calculated for all dependent measures. Then between-group differences were further calculated for each measure (e.g., between-group difference in *f0*-correlation change = *f0*-correlation change in music-intervention group – *f0*-correlation change in language-experience group). This process was repeated 1000 times and distributions of permuted between-group differences can be visualized for each measure in Figure 3C, with each row for each dependent measure. Corresponding ASL values were calculated as the proportion in the permutation distribution where the permuted group difference is more extreme than the observed group difference.

## Results

### Quality of pitch tracking declines in the language-experience group

For the language-experience group, the observed pitch contour (*f0*) for each age, extracted from the FFRs, can be visualized in Figure 2A. The pitch contour (*f0*) extracted from the stimulus using the same method is also shown. Pitch tracking of the non-native lexical tone in the language-experience group was found to be highly similar to the actual stimulus pitch contour at 7 months of age. However, at 11 months of age, infants’ FFR pitch tracking is much less precise, as predicted by perceptual narrowing. This difference is especially remarkable in the first half of the stimulus signal.

In Figure 3A, the distributions of permuted *between-age* differences in the language-experience group can be visualized, with each row for each dependent measure. The observed differences can be visualized by the red lines. The ASL for the between-age difference in the main pitch tracking quality metric (i.e., *f0-*correlation) is 0.029. Additionally, the ASL is 0.055 for *f0-*error, 0.686 for *f0-*strength and 0.628 for SNR. The comparable SNR between the two ages indicates that the developmental differences observed in *pitch-*related measures are unlikely to be contributable to differences in SNR.

### Quality of pitch tracking is maintained in the music-intervention group

The observed pitch contour (*f0*) for each age, extracted from the FFRs, in the music-intervention group can be visualized in Figure 2B. As shown, infant’s FFR pitch tracking of the non-native lexical tone is largely comparable at 7 and 11 months-of-age. Infants’ pitch-tracking abilities are thus maintained at 11 months for the infants who underwent the music intervention.

In Figure 3B, the distributions of permuted *between-age* differences in the music-intervention group can be visualized, with each row for each dependent measure. The observed differences can be visualized by the red lines. The ASL for between-age difference in the main metric *f0*-correlation is 0.148. Additionally, the ASL is 0.516 for *f0*-error, 0.606 for *f0*-strength and 0.088 for SNR.

### Change in pitch tracking quality between 7 and 11 months differed between the two groups

In Figure 3C, the distributions of permuted *between-group* differences can be visualized, with each row for each dependent measure. The observed differences can be visualized by the red lines. The ASL for *f0*-correlation change between the two groups is 0.031. Additionally, the ASL for between-group difference is 0.086 for change in *f0*-error, 0.366 for change in *f0*-strength and 0.104 for change in SNR.

## Discussion

The current study addressed theoretically important questions regarding the neural mechanisms that underlie the sensitive period for phonetic learning in infants, one of the earliest and most important milestones in early language acquisition. Specifically, we examined whether early sensory encoding of speech is altered by experience during this very early period in development. We tested two hypotheses regarding this question. First, we tested the hypothesis that early sensory encoding of speech exhibits a decline for non-native speech during the sensitive period, reflecting the perceptual narrowing phenomenon related to increased native language experience. Second, we tested the hypothesis that music intervention during this period can reverse the decline and maintain the auditory systems’ ability to track acoustic properties of non-native speech. This would bolster the hypothesis that during this early period of development, music experience can already strengthen the shared neural mechanisms for music and speech processing.

We measured monolingual English-learning infants’ FFR to a non-native Mandarin lexical tone longitudinally at both 7 and 11 months of age. Between the two FFR measurements, infants were randomly assigned to receive either no intervention (language-experience group) or a 12-session music intervention (music-intervention group). The FFR revealed the precision with which infants neurally tracked the pitch of the non-native Mandarin tone. The results showed that (1) the precision of neural pitch tracking indeed declined in the language-experience group, as expected due to the perceptual narrowing phenomenon, (2) the precision of neural pitch tracking was maintained with age in the music-intervention group, thus reversing the decline seen with the language-experience group, and (3) the change in neural pitch tracking from 7 to 11 months was different between the two groups. Our results therefore support both study hypotheses, suggesting that auditory experience in infants can affect their sensory encoding of speech in early stages of auditory processing.

The current results expand our current theoretical understanding of the neural mechanisms underlying the sensitive period for phonetic learning. To date, research has focused on the MMN/MMR, which reflects later and higher-level neural processes. The current study instead focuses on the precision of sensory encoding of speech by examining the representation of the stimulus early in the auditory processing stages. Indeed, the results demonstrated that the effect of early auditory experience is pervasive; it extends to early encoding stages in the auditory system (i.e., FFR) very early in development (i.e., in infancy), and it demonstrates malleability with relatively short periods of additional experience (i.e., a 4-week music intervention). These results complement existing literature on adults and older children with vastly different early experiences (e.g., linguistic background, music training background) demonstrating differences in early sensory encoding (Wong et al., 2007; Kraus et al., 2014; Zhao and Kuhl, 2018), and suggest that these experiential effects may start accumulating during infancy. However, it is also important to note that the auditory system is also undergoing rapid maturation process during this period (Anderson et al., 2015; Novitskiy et al., 2021), and it is important for future research to disentangle the interaction between maturation and speech learning at the sensory encoding stage, by implementing additional conditions including native and non-native speech as well as non-speech stimuli.

The current results also provide new evidence supporting the idea of shared neural mechanisms for speech and music processing in infancy (Patel, 2011, 2014), that is, speech processing is enhanced when underlying shared neural mechanisms become strengthened by music intervention. Previous research has demonstrated that the same short-term laboratory controlled music intervention during the sensitive period can indeed generalize from the music domain to the speech domain (i.e., cross-domain generalization), by focusing on the MMR (Zhao and Kuhl, 2016). The current result replicates and extends the previous finding, demonstrating that generalization effects from music intervention to speech are not limited to later and higher-level neural processes, but are also observed in neural representation of acoustic features (e.g., pitch) in speech at the earlier stages of auditory processing.

A few caveats should be noted in this current study. First, while the current stimulus for FFR recording (i.e., /yi/ in tone 3) was selected given it was optimal for demonstrating language-experience effects in adults (Llanos et al., 2017), it is still possible that the experiential effects (i.e., language and music) may manifest differently for other lexical tones and/or other speech sounds (e.g., consonants, vowels). Indeed, previous research has documented other developmental patterns for different speech sounds during the sensitive period (Best et al., 2016), and music intervention may affect different speech sounds (e.g., other lexical tones, consonants, vowels) differently. Thus, future replication and expansion using various speech sounds in both native and non-native languages are thus warranted for a more comprehensive understanding of the experiential effects on infants’ speech learning.

Second, the language-experience group in the current study did not participant in any lab-sessions in between their FFR measurements. While this group allows accurate assessment of the effect of language experience in typically developing monolingual English-learning infants (i.e., language-experience group), it does not allow us to isolate effects of music intervention from activate lab visits for the music-intervention group. Given that the previous study using the exact same music intervention with an active control group (i.e., control group participated in same number of free play sessions in the lab) demonstrated a transfer effect on speech discrimination (Zhao and Kuhl, 2016), we concluded that the effects in the current study should also be largely from the music intervention. Future replication is needed and additional levels of controls (e.g., free play, passive music listening) are warranted to further elucidate the relative contribution of different components of the music intervention to the generalization effect, such as the movement or the social engagement. Further research is also needed to allow for a comprehensive understanding of the effects of music intervention on language development and whether the effects are similar for infants at risk for communication disorders as it is for typically developing infants. Understanding these questions will move us closer to an understanding of how effective early music intervention may be as an early intervention tool for communication disorders.

## Data availability statement

The datasets generated and analyzed for this study can be found in the Open Science Framework (https://osf.io/4s9h8/?view_only=ad50d59987314412a81626eaf9390fa9). The music intervention group was registered on the clinicaltrials.gov under trial number NCT04509739.

## Ethics statement

The studies involving human participants were reviewed and approved by University of Washington Institute Review Board. Written informed consent to participate in this study was provided by the participants or their legal guardian/next of kin.

## Author contributions

TCZ and PKK: conceptualization. TCZ, FL, and BC: methodology. TCZ and FL: visualization. BC and PKK: supervision. TCZ: investigation and writing—original draft. FL, BC, and PKK: writing—review and editing. All authors contributed to the article and approved the submitted version.
